# Neural Dynamics Underlying the Evaluation Process of Ambiguous Options During Reward-Related Decision-Making

**DOI:** 10.3389/fpsyg.2019.01979

**Published:** 2019-08-28

**Authors:** Chengkang Zhu, Jingjing Pan, Yiwen Wang, Jianbiao Li, Pengcheng Wang

**Affiliations:** ^1^Reinhard Selten Laboratory, Business School, China Academy of Corporate Governance, Nankai University, Tianjin, China; ^2^School of Economics, Institute for Study of Brain-like Economics, Shandong University, Jinan, China; ^3^China Center of Social Trust Research, Fuzhou University, Fuzhou, China; ^4^Department of Economic and Management, Nankai University Binhai College, Tianjin, China; ^5^Business School, Tianjin University of Economic and Finance, Tianjin, China

**Keywords:** ambiguous options, evaluation, neural dynamics, P3, delta activity

## Abstract

Ambiguous decision-making involves different processes. However, few studies have focused on the evaluation process. In this study, event-related potentials (ERPs) and event-related spectrum perturbation (ERSP) techniques were used to explore the neural dynamics underlying the evaluation process of ambiguous options through an ambiguous choice task. Some important results emerged. We found a preference for lotteries with low ambiguity regardless of reward amount, suggesting that subjects were averse to ambiguity in our paradigm. Our electroencephalography (EEG) results clarified the neural dynamics underlying the evaluation process. In the time domain, lotteries with both a larger reward and lower ambiguity elicited a larger P3. In the time-frequency domain, larger amplitudes of delta activity at 200–400 ms and 500–600 ms post-stimulus were elicited by lotteries with low ambiguity. Moreover, lotteries with a larger reward elicited larger amplitudes of delta activity at 400–600 ms post-stimulus. Our ERPs and ERSP results suggested that individuals in our paradigm evaluated ambiguity and reward separately, and then integrated their evaluation to form subjective values of different lotteries.

## Introduction

Decision-making under uncertainty permeates our daily life. According to the precise likelihood of outcome, economists divide two types of uncertain events: risk and ambiguity. For risk, the outcome probability corresponds to a point estimation. For ambiguity, the outcome probability is either unknown (Ellsberg, 1961) or an interval estimation (Becker and Brownson, 1964; Curley and Yates, 1985; Rustichini et al., 2005). The majority of uncertainty in real situations is ambiguity. In many experiments related to ambiguity, one of the most prominent phenomena, referred to as “ambiguity avoidance,” is the individual’s preference for risk over ambiguity (Ellsberg, 1961; Camerer and Weber, 1992). Decision-making is a continuous process, which entails the evaluation of ambiguous options, formation of preference, choice, and learning from feedback (Wang et al., 2015). Ambiguity avoidance has been demonstrated to emerge during the evaluation process (Rode et al., 1999). During the evaluation of ambiguous options, the mean outcome and its variance are integrated to form preference. Ambiguity is supposedly averse because of its high outcome variance. However, this speculation lacks support from neural dynamic evidence of the evaluation of ambiguous options.

The focus of previous neuroimaging studies has been on contrasting the neural mechanism related to decision-making under risk and ambiguity. Decision-making under ambiguity elicits greater activity in the amygdala, orbitofrontal cortex, lateral prefrontal cortex, anterior insular cortex, posterior inferior frontal gyrus, and posterior parietal cortex, and less activity in the striatum (Hsu et al., 2005; Huettel et al., 2006; Bach et al., 2009). One functional magnetic resonance imaging (fMRI) study investigated the neural representation of subjective value under risk and ambiguity (Levy et al., 2010). In that study, subjects were asked to make decisions under different levels of risk and ambiguity. Their behavioral data were used to calculate the subjective value of each option, and neural activity was measured. The results revealed that the activities of the striatum, medial prefrontal cortex, posterior cingulate cortex, and amygdala were correlated with the subjective value of risky and ambiguous options.

Event-related potentials (ERPs) and event-related spectral perturbations (ERSPs) have millisecond-level temporal resolution, which is useful in exploring the evaluation process of ambiguous options. Existing ERP studies have been mainly focused on neural correlates underlying the choice and feedback stage of decision-making under uncertainty (for reviews, see Chandrakumar et al., 2018). To our knowledge, only one ERP study has explored the neural mechanism underlying the evaluation stage (Wang et al., 2015). In their experiment, participants were asked to decide whether to bet or not, under ambiguity and risk. They made decisions after a random monetary reward was presented. They would either earn or lose the monetary reward if they decided to bet. Otherwise, they would earn nothing. The results revealed that a larger P3 was elicited by risky options compared with ambiguous options. Previous ERP studies have shed light on the neural dynamics of decision-making under ambiguity (Gu et al., 2010; Xu et al., 2011; West et al., 2014; Kóbor et al., 2015; Mussel et al., 2015; Wang et al., 2015; Endrass et al., 2016; Azcárraga-Guirola et al., 2017). However, there have been limitations. Decision-making under ambiguity includes several stages, from the evaluation of ambiguous options to feedback processing. Few ERP studies have been focused on the evaluation process. Although Wang et al. (2015) explored the neural dynamics underlying the evaluation of ambiguous options, they mainly aimed at comparing the neural mechanism of ambiguity and risk. Their paradigm did not allow one to distinguish between evaluation and choice processes. Moreover, their study did not clarify the temporal dynamics of ambiguous option evaluation, which entails processing of the level of ambiguity, reward amount, and the corresponding integration process.

In this study, we used ERPs and the ERSP technique to investigate neural temporal dynamics underlying the process of ambiguous option evaluation. Therefore, we developed an ambiguous choice task. Our task paradigm was derived from previous literature on risky choice (Wang et al., 2019), given that the evaluation of ambiguous options is somewhat similar to that of risky options (Levy et al., 2010). In our task, two ambiguous lotteries were serially presented. Subjects were then asked to choose one lottery to decide their payoff. This allowed us to separate evaluation and choice processes. No feedback was shown to the subjects to control for the learning effect. By varying the probability interval of reward, we manipulated the level of ambiguity, based on the methods of Levy et al. (2010). We set up four types of lotteries: high ambiguity with a reward of 20 Chinese yuan (CNY) (H20); high ambiguity with a reward of 10 CNY (H10); low ambiguity with a reward of 20 CNY (L20); and low ambiguity with a reward of 10 CNY (L10). For each reward, lotteries with different levels of ambiguity led to the same mean reward. Using this paradigm, we were able to clarify the integration process of ambiguity and mean reward during the evaluation of an ambiguous option.

Several electroencephalography (EEG) components in the time domain and time-frequency domain can be used to explore the process of ambiguous option evaluation. In the time domain, the relevant component during the evaluation stage is P3 (Goldstein et al., 2006; Broyd et al., 2012; Zhang et al., 2017; Zheng et al., 2017). The P3 peak at 300–600 ms post-stimulus at posterior scalp sites is associated with reward evaluation and anticipation (Pfabigan et al., 2015). Furthermore, P3 possibly encodes the subjective value of each ambiguous option. Thus, we hypothesized that both low ambiguity and reward of 20 CNY would elicit larger P3 amplitudes. In the time-frequency domain, delta power (1–4 Hz) is an index of reward processing (for review, see Knyazev, 2007, 2012). Therefore, the dynamics of delta activity when faced with four types of lotteries would reflect the sequential order of processing the ambiguity, mean reward, and integration between the two. We predicted that low ambiguity would elicit a larger delta band activity when processing the ambiguity, while the reward of 20 CNY would elicit a larger delta band activity when processing the mean reward. Moreover, we predicted that both low ambiguity and the reward of 20 CNY elicited larger delta band activity when processing the integration between ambiguity and mean reward.

## Materials and Methods

### Participants

A total of 25 healthy volunteers (age range = 21–25 years; females = 12) from Nankai University participated in this study. Sample size was determined by power analysis. All participants were right-handed, native Chinese speakers. The participants had normal or corrected-to-normal vision and no history of psychiatric or neurological disorders. Each participant signed written informed consent forms and received a base payment of 30 Chinese yuan (CNY, roughly equal to US $4.50) for participation, plus a bonus of 0–60 CNY based on his/her decision. The study protocol was approved by the Ethics Committee of Nankai University. The procedures were performed in accordance with approved guidelines and the Declaration of Helsinki. Materials and data related to this study will be made available upon request.

### Stimuli and Task

**Figure 1:**
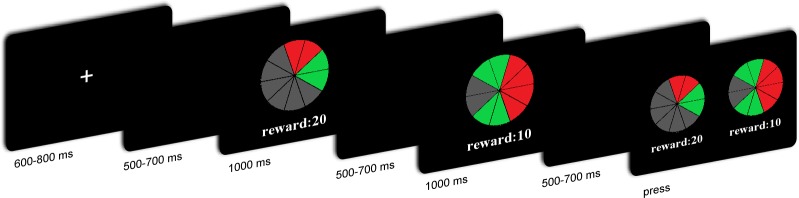
Overview of the task and trial structure.

The participants performed an ambiguous choice task. In each trial, participants were presented with two lotteries sequentially with varying ambiguity levels and varying reward amounts. Participants had to indicate which lottery they preferred. Each lottery appeared on the screen in the form of a “pie” painted partly red and partly green (Figure 1). All pies contained 10 sectors. Participants were told that each image on the screen represented a physical bag containing 10 balls. The relative numbers of red and green balls were indicated by the proportions of red and green sectors. Part of the pie was hidden by a gray occluder. The probability of drawing green or red balls was therefore ambiguous. Two different occluder sizes (covering either two or six sectors) represented two ambiguity levels. For the low ambiguity level, the probability of drawing a red ball could have been anywhere between 40 and 60%. Similarly, the probability of drawing a green ball could have been anywhere between those two values. For the high ambiguity level, the probability of drawing a red ball could have been anywhere between 20 and 80%. The probability of drawing a green ball could also have been anywhere between 20 and 80%. The number under the pie represented the amount of money to be won that was associated with the target color. For half of the participants, drawing a red ball yielded a given amount of money and drawing a green ball yielded nothing. For the other half of the participants, drawing a green ball yielded a given amount of money, and a red ball yielded nothing. Two reward amounts (10 and 20 CNY) were used at each ambiguity level, to give four types of lotteries, i.e., H20, H10, L20, and L10.

At the beginning of the experiment, participants were told that each lottery corresponded with a unique bag. Therefore, we provided four sealed bags associated with four types of lotteries. Before the task, participants were asked to sign their names on the sealed bags. This method was used to ensure the relative numbers of red and green balls could not have been adjusted by experimenters during the task. At the end of the experiment, three trials were randomly selected by computers. Based on the lotteries they chose in these trials, participants then drew a ball from each corresponding bag (if two or three lotteries were the same, they would draw a ball twice or three times from the corresponding bag with replacements). In addition to their participation fee, they were paid according to the lotteries and number of balls of the target color.

### Procedure

The EEG recording was performed in a small, sound-attenuated, electrically shielded chamber. After the EEG electrodes were attached, the participants sat in a comfortable chair that was approximately 100 cm in front of a 23-inch (58.42 cm) computer monitor. Before the tasks began, all participants read the instructions carefully and were asked to take eight practice trials. Figure 1 shows the timeline of a single trial. Each trial began with the presentation of a single centrally located white fixation cross for 600–800 ms. A black screen was then presented for 500–700 ms, followed by the first lottery for 1,000 ms. Subsequently, the second lottery was presented for 1,000 ms, after which, a black screen was presented for 500–700 ms. The order of these lotteries was counterbalanced. Thereafter, participants were asked to choose one of the lotteries to decide their payoff in that trial.

The entire experiment comprised 80 test and eight practice trials. Only the test trials were used for EEG analysis. The trials occurred within four blocks of 20 trials. Each block was separated by a break, the duration of which was determined by the participants. All 80 trials were performed within 15–25 min, during which the trials were randomly presented. The E-Prime software was used to control the display of stimuli and acquisition of behavioral data (Version 2.0, Psychology Software Tools, Inc.).

### Electroencephalography Acquisition

The EEG data were recorded continuously with a 40-channel NuAmps DC amplifier (Compumedics Neuroscan, Inc., Charlotte, NC, USA). According to the International 10-20 System, 32 active Ag/AgCl electrodes were used. The EEG was sampled at 1,000 Hz using a 22-bit A/D converter. The reference and ground electrodes were positioned at AFz, and the impedances of all electrodes were kept below 10 kΩ.

### Electroencephalography Analysis

Preprocessing of EEG data was performed with the EEGLAB 14.1.1 tool (Delorme and Makeig, 2004), implemented in MATLAB 2017a. In addition, a 0.1/30 Hz high-/low-pass filter was applied after the reference of EEG signals was reset to the average of the left and right mastoids. Individual epochs were extracted from −1,000 to 2,000 ms around the presentation of the stimulus defined as the lotteries that sequentially presented in our task. A manual artifact correction procedure was applied to eliminate trials with artifacts, based on visual inspection. Independent component analysis (ICA) was performed to remove eye movement, and the related ICA components were manually selected. Artifact-free epochs of each subject were grouped into four conditions, i.e., H20, H10, L20, and L10.

Clean EEG data were analyzed in the time domain. The 1,000-ms epochs were extracted, starting at 200 ms before the presentation of the stimulus. A 200-ms pre-stimulus period was used as baseline, and the accepted epochs were baseline-corrected. The P3 was scored as the mean voltage from 500 to 600 ms post-stimulus at Pz, corresponding to the 100-ms time window surrounding the peak.

Time-frequency analysis was performed using the Fieldtrip toolbox (Oostenveld et al., 2011) built-in ft_freqanalysis function, based on complex Morlet wavelet convolution (1–10 cycles, 1–30 Hz, 120 spaced frequencies, 1,000 time points per epoch). The time interval of −200 to 0 ms before presentation of the stimulus was used for baseline normalization. The mean converted amplitudes within 1–4 Hz from 200 to 300 ms, 300 to 400 ms, 400 to 500 ms, and 500 to 600 ms at Pz were used to analyze the delta band power change in different time windows.

For all analyses, the values of *p* were corrected using the Greenhouse-Geisser correction when the sphericity assumption was violated. A value of *p* < 0.05 was considered significant. Significant interaction was analyzed using the simple effect model. Statistics were analyzed using the SPSS 19.0 software.

## Results

### Behavior Data

**Figure 2:**
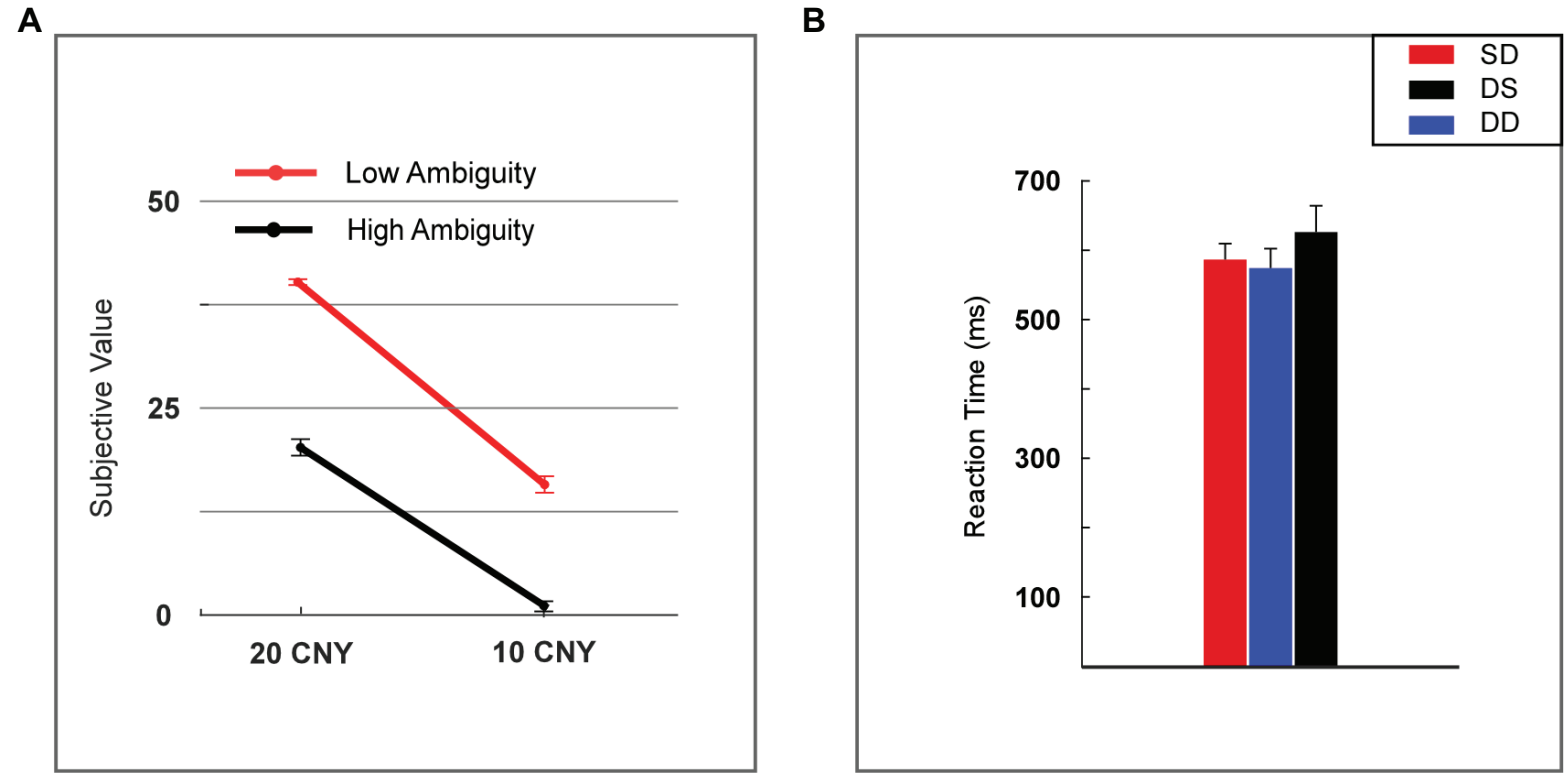
Behavioral results. **(A)** Subjective value among lotteries (H20, H10, L20, and L10). **(B)** Reaction time among lotteries (H20, H10, L20, and L10). Error bars represent SEM. H20, high ambiguity with a reward of 20 CNY; H10, high ambiguity with a reward of 10 CNY; L20, low ambiguity with a reward of 20 CNY; L10, low ambiguity with a reward of 10 CNY. SD, two lotteries with same ambiguity level but different reward amounts; DS, two lotteries with different ambiguity levels but same reward amount; DD, two lotteries with different ambiguity levels and different reward amounts.

The subjective value of a lottery was defined as the frequency with which it was selected by the participants. The subjective value was analyzed using two-way repeated measures ANOVA (rmANOVA) with ambiguity levels (high vs. low) and reward amounts (10 vs. 20 CNY) as within-subject factors (Figure 2A). A significant main effect was found for reward amount [*F*(1, 24) = 452.859, *p* < 0.001, partial *η*^2^ = 0.950], as a higher subjective value was noted for 20 CNY (mean ± SEM = 30.860 ± 1.392) versus 10 CNY (mean ± SEM = 9.140 ± 1.119). A significant main effect was also found for ambiguity [*F*(1, 24) = 228.553, *p* < 0.001, partial *η*^2^ = 0.905], as a higher subjective value was noted for low ambiguity (mean ± SEM = 27.840 ± 1.808) versus high ambiguity (mean ± SEM = 12.160 ± 1.492). A significant interaction effect was revealed [*F*(1, 24) = 15.600, *p* = 0.001, partial *η*^2^ = 0.394]. For high ambiguity, a significant main effect was observed with reward amount [*F*(1, 24) = 210.251, *p* < 0.001, partial *η*^2^ = 0.898], as a higher subjective value was noted for 20 CNY (mean ± SEM = 21.760 ± 1.007) than 10 CNY (mean ± SEM = 2.560 ± 0.663). For low ambiguity, a significant main effect was also observed with reward amount [*F*(1, 24) = 513.497, *p* < 0.001, partial *η*^2^ = 0.955], as a higher subjective value was observed for 20 CNY (mean ± SEM = 39.960 ± 0.040) than 10 CNY (mean ± SEM = 15.720 ± 1.053). For 20 CNY, a significant main effect was observed with ambiguity level [*F*(1, 24) = 334.586, *p* < 0.001, partial *η*^2^ = 0.933], as a higher subjective value was noted for low ambiguity (mean ± SEM = 39.960 ± 0.040) than high ambiguity (mean ± SEM = 21.760 ± 1.007). For 10 CNY, a significant main effect was also observed with ambiguity level [*F*(1, 24) = 87.662, *p* < 0.001, partial *η*^2^ = 0.785], as a higher subjective value was noted for low ambiguity (mean ± SEM = 15.720 ± 1.053) than high ambiguity (mean ± SEM = 2.560 ± 0.663).

Reaction time was analyzed using one-way rmANOVA with conditions (two lotteries with the same ambiguity level but different reward amounts vs. two lotteries with different ambiguity levels but same reward amount vs. two lotteries with different ambiguity levels and different reward amounts, henceforth referred to as SD vs. DS vs. DD). We found no significant main effects [*F*(2, 48) = 1.818, *p* = 0.184, partial *η*^2^ = 0.070] for the various conditions (Figure 2B).

### Electrophysiological Data

#### P3

**Figure 3:**
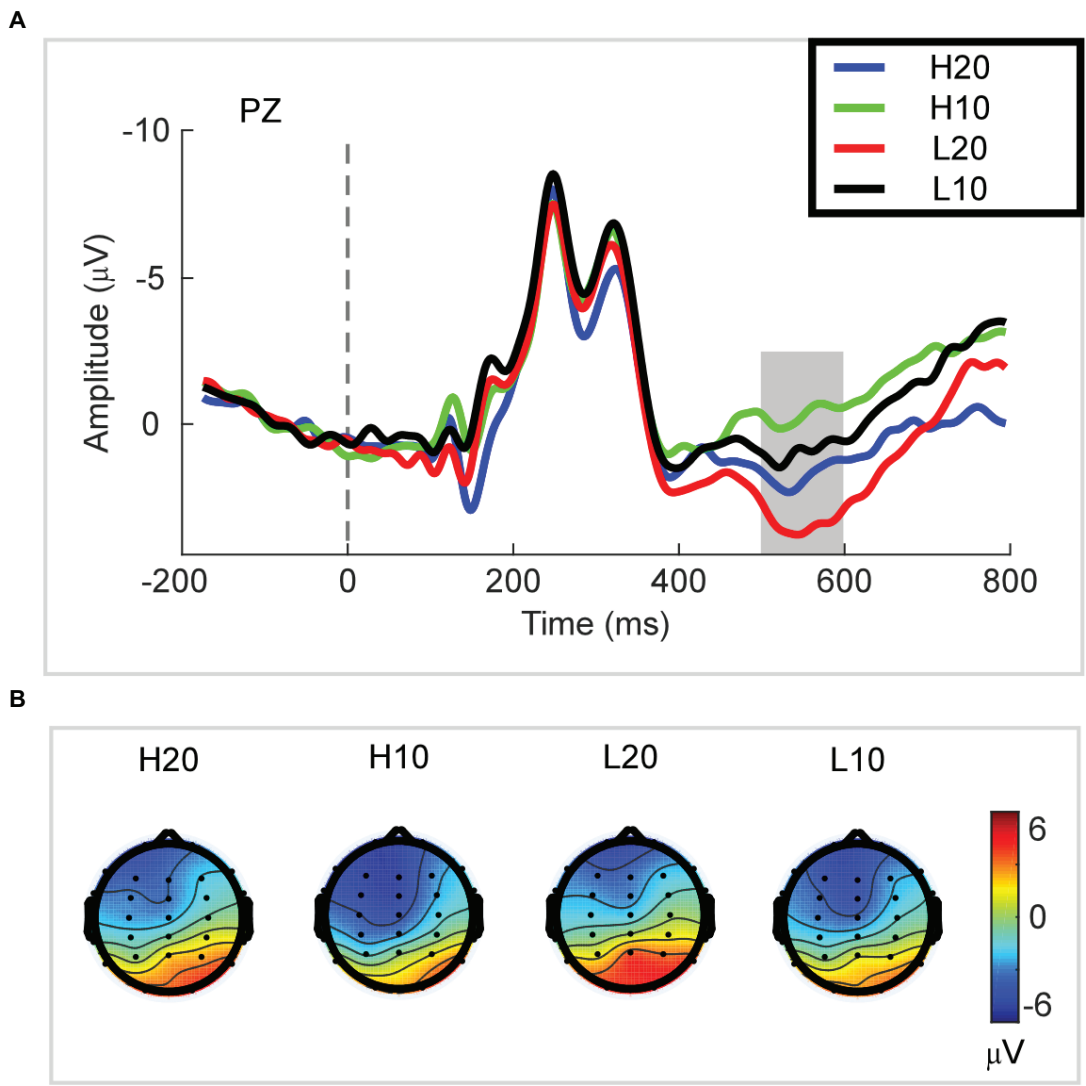
P3 results. **(A)** Grand average ERP waves computed at Pz. **(B)** Topographic voltage maps of mean amplitude of the P3 wave.

A two-way rmANOVA was performed with ambiguity (high vs. low) and reward (10 vs. 20 CNY) as factors (Figure 3). A significant main effect was found [*F*(1, 24) = 33.891, *p* < 0.001, partial *η*^2^ = 0.585] for reward amount, with a larger amplitude of P3 for 20 CNY (mean ± SEM = 2.730 ± 0.376 μV) than 10 CNY (mean ± SEM = 0.403 ± 0.417 μV). Moreover, a significant main effect was also found [*F*(1, 24) = 12.367, *p* = 0.002, partial *η*^2^ = 0.340] for ambiguity level, with a larger amplitude of P3 for low ambiguity (mean ± SEM = 2.283 ± 0.430 μV) than high ambiguity (mean ± SEM = 0.824 ± 0.373 μV). However, no significant interaction effects were found.

#### Delta Activity

**Figure 4:**
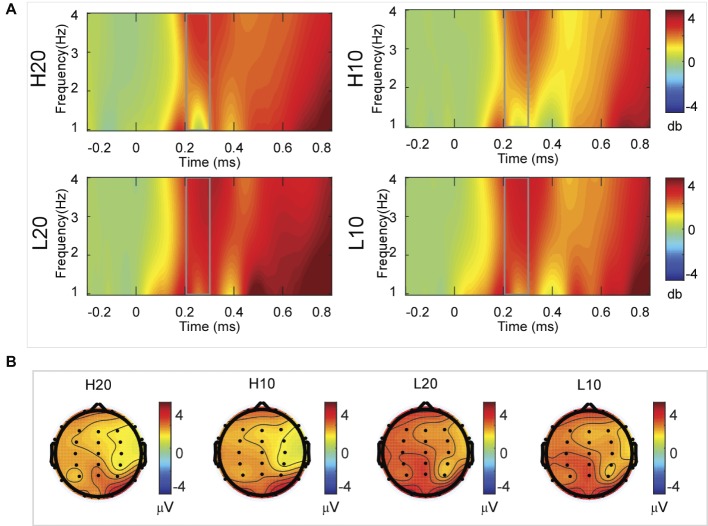
Delta results from 200 to 300 ms. **(A)** Delta (1–4 Hz) activity at Pz. **(B)** Topographic maps of the mean amplitude of delta band power within 1–4 Hz from 200 to 300 ms.

A two-way rmANOVA was performed on the delta power from 200 to 300 ms with ambiguity (high vs. low) and reward (10 vs. 20 CNY) as factors (Figure 4). Only one significant main effect was found [*F*(1, 24) = 11.822, *p* = 0.002, partial *η*^2^ = 0.330] for ambiguity level, with a larger amplitude for low ambiguity (mean ± SEM = 2.379 ± 0.259 dB) than high ambiguity (mean ± SEM = 1.733 ± 0.218 dB). However, we found no significant main effects for reward or significant interaction effects.

**Figure 5:**
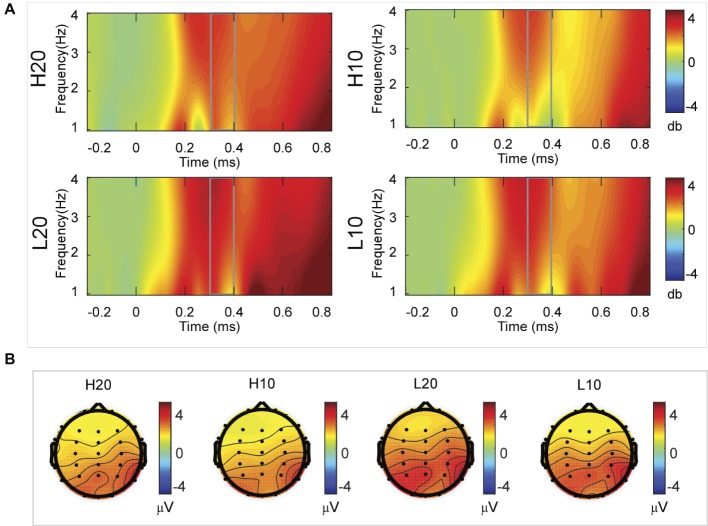
Delta results from 300 to 400 ms. **(A)** Delta (1–4 Hz) activity at Pz. **(B)** Topographic maps of the mean amplitude of delta band power within 1–4 Hz from 300 to 400 ms.

A two-way rmANOVA was performed on the delta power from 300 to 400 ms with ambiguity (high vs. low) and reward (10 vs. 20 CNY) as factors (Figure 5). Only one significant main effect was found [*F*(1, 24) = 7.646, *p* = 0.011, partial *η*^2^ = 0.242] for ambiguity, with a larger amplitude for low ambiguity (mean ± SEM = 2.444 ± 0.255 dB) than high ambiguity (mean ± SEM = 1.864 ± 0.219 dB).

**Figure 6:**
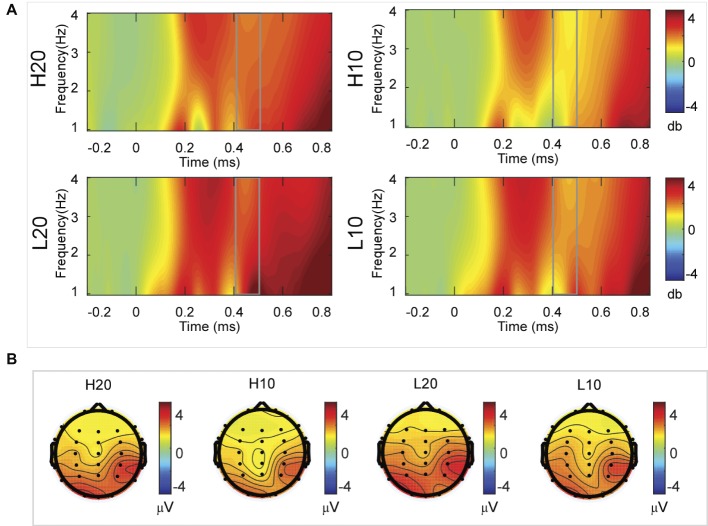
Delta results from 400 to 500 ms. **(A)** Delta (1–4 Hz) activity at Pz. **(B)** Topographic maps of the mean amplitude of delta band power within 1–4 Hz from 400 to 500 ms.

A two-way rmANOVA was performed on the delta power from 400 to 500 ms with ambiguity (high vs. low) and reward (10 vs. 20 CNY) as factors (Figure 6). Only one significant main effect was found [*F*(1, 24) = 9.846, *p* = 0.004, partial *η*^2^ = 0.291] for reward amount, with a larger amplitude for 20 CNY (mean ± SEM = 2.252 ± 0.200 dB) than 10 CNY (mean ± SEM = 1.502 ± 0.194 dB).

**Figure 7:**
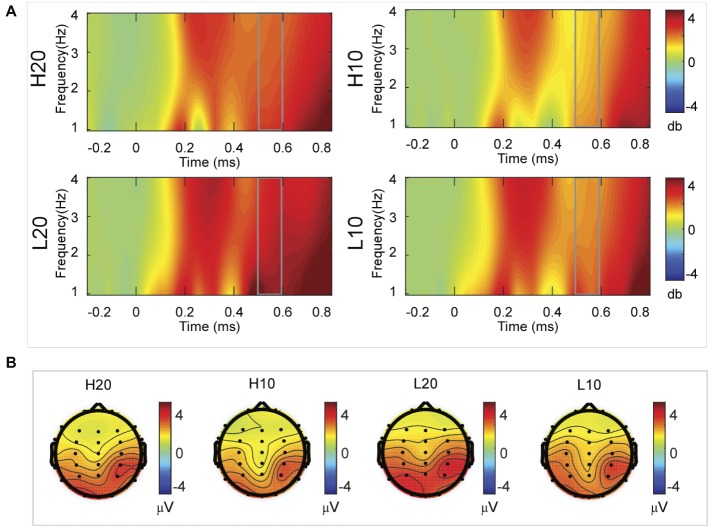
Delta results from 500 to 600 ms. **(A)** Delta (1–4 Hz) activity at Pz. **(B)** Topographic maps of the mean amplitude of delta band power within 1–4 Hz from 500 to 600 ms.

A two-way rmANOVA was performed on the delta power from 500 to 600 ms with ambiguity (high vs. low) and reward (10 vs. 20 CNY) as factors (Figure 7). A significant main effect was found [*F*(1, 24) = 6.772, *p* = 0.016, partial *η*^2^ = 0.220] for ambiguity level, with a larger amplitude for low ambiguity (mean ± SEM = 2.485 ± 0.212 dB) than high ambiguity (mean ± SEM = 1.854 ± 0.160 dB). Moreover, a significant main effect was found [*F*(1, 24) = 13.481, *p* = 0.001, partial *η*^2^ = 0.360] for reward, with a larger amplitude for 20 CNY (mean ± SEM = 2.658 ± 0.191 dB) than 10 CNY (mean ± SEM = 1.681 ± 0.199 dB). However, no significant interaction effects were found.

## Discussion

Ambiguous decision-making involves different processes, from evaluation to feedback. Previous behavioral studies have suggested that ambiguity aversion occurs because of a lower subjective value with high variance of the mean outcome during the evaluation process. However, this speculation lacks supportive evidence from neural dynamics analyses. In this study, ERP and ERSP techniques were used to explore the neural dynamics underlying the evaluation process of ambiguous options through an ambiguous choice task. Some important results have emerged. We found a preference for lotteries with low ambiguity regardless of reward amount, suggesting that subjects were averse to ambiguity in our paradigm. Our EEG results clarified the neural dynamics underlying the evaluation process. In the time domain, both lotteries with larger rewards and those with low ambiguity elicited a larger P3. In the time-frequency domain, larger amplitudes of delta activity at 200–400 and 500–600 ms post-stimulus were elicited by lotteries with low ambiguity. Moreover, lotteries with larger rewards elicited a larger amplitude of delta activity at 400–600 ms post-stimulus.

Our behavioral data showed that most participants disliked lotteries with high ambiguity and small rewards. This finding is consistent with previous studies regarding decision-making under ambiguity (Rode et al., 1999; Levy et al., 2010). In our task, the subjective value of lottery H10 was 2.56. When the ambiguity was low, the subjective value increased to 15.72. As the reward increased to 20 CNY, the subjective value increased significantly and reached 21.76 in cases of high ambiguity. Moreover, for lottery L20, the subjective value was 39.96 and significantly higher than all other lotteries. Lower ambiguity led to an increased frequency with which the lottery was chosen, and therefore, a larger subjective value. We noted that the reaction time was identical among different choice conditions (i.e., SD vs. DS vs. DD). This result suggested that the difficulty of choosing between different types of lotteries was similar in our task.

In the time domain, we observed an obvious P3 component peaking at approximately 500–600 ms following the presentation of the lotteries on the screen. This component reflects the stimulus categorization process (for reviews, see Polich, 2007) and motivational salience to the stimulus (for review, see Polich and Kok, 1995). The P3 wave has also been associated with activation of the ventral striatum (Pfabigan et al., 2015) during the evaluation process. Since the ventral striatum is a region related to reward processing (Delgado et al., 2000; Schultz, 2000; Breiter et al., 2001; Knutson et al., 2003; Tobler et al., 2007; Haber and Knutson, 2010; Kahnt et al., 2011; Sescousse et al., 2013, 2014; Wilson et al., 2018), the P3 can be an index of reward evaluation. In our study, both lotteries with low ambiguity and those with larger rewards elicited a larger amplitude of P3, indicating that P3 integrated the evaluation of ambiguity and reward. Among four types of lotteries, the amplitude of P3 for H10 was the smallest at only −0.215 μV. When the ambiguity was low, the amplitude of P3 increased to 1.022 μV. As the reward increased to 20 CNY, the amplitude of P3 increased and reached 1.863 μV in cases of high ambiguity. For the L20 lotteries, the amplitude of P3 was 3.543 μV, the highest among all lotteries. Considering our behavioral and ERP data together, we found that a larger amplitude of P3 led to an increased frequency with which a lottery was selected. Thus, we suggested that the amplitude of P3 during the evaluation process encoded the subjective value of each ambiguous lottery, and could be used to predict the subsequent choice.

In the time-frequency domain, delta activity is sensitive to reward evaluation during reward anticipation processing (for review, see Knyazev, 2007, 2012; Glazer et al., 2018). It could also be an index representing the integration of reward (Gheza et al., 2018; Zhu et al., 2019). In the present study, the dynamics of delta power for lotteries during the evaluation process reflected the subjects’ processing of the elements of the lotteries (i.e., ambiguity level and mean reward) and their integration. At 200–400 ms after the stimulus, lotteries with low ambiguity elicited larger amplitudes of delta activity, indicating that individuals started to evaluate ambiguity at about 200 ms after the presentation of lotteries on the screen. This result also suggested that individuals preferred lotteries with low ambiguity to those with high ambiguity. During the next 100 ms, lotteries with a larger reward elicited larger amplitudes of delta activity, indicating that individuals evaluated reward at 400–500 ms post-stimulus. Moreover, this result suggested that individuals preferred larger rewards. At 500–600 ms post-stimulus, both lotteries with low ambiguity and those with larger rewards elicited larger amplitudes of delta activity. These results indicated that subjects integrated their evaluation of ambiguity and reward to form a subjective value of lotteries at 500–600 ms after presentation of the lotteries. This supports our findings in the time domain and supports the idea that delta activity plays a key role in P3 generation during reward evaluation (Demiralp et al., 2001; Bernat et al., 2007, 2015; Ergen et al., 2008; Ishii et al., 2009).

In summary, to our knowledge, this study is the first to investigate the neural dynamics underlying the evaluation process of lotteries under ambiguity. Our ERPs and ERSP results suggest that individuals in our paradigm evaluated ambiguity and reward separately. The ambiguity level was evaluated at 200–400 ms and the reward was evaluated at 400–500 ms after the lotteries were presented on the screen. At 500–600 ms after the stimulus, individuals integrated the evaluation of ambiguity and reward to form a subjective value of the different lotteries. These findings shed light on our understanding of the temporal course of processing ambiguous options. Furthermore, our findings provide neural dynamic evidence of the emergence of ambiguity avoidance during the evaluation process. One limitation of this study should be mentioned. Although the evaluation of ambiguity was earlier than that of reward in our task, whether individuals in other tasks evaluate ambiguity first is unclear. Future studies should explore the impacting factor of evaluation sequence during the process of ambiguous decision-making.

## Data Availability

The datasets generated for this study are available on request to the corresponding author.

## Ethics Statement

This study was carried out in accordance with the recommendations of the Ethics Committee of Business of Nankai University committee with written informed consent from all subjects. All subjects gave written informed consent in accordance with the Declaration of Helsinki. The protocol was approved by the Ethics Committee of Business of Nankai University committee.

## Author Contributions

CZ, JP, PW, and JL designed the experiment. CZ and JP carried out the experiment, analyzed the data, wrote the paper, and contributed equally to this work. CZ, JP, JL, YW, and PW revised the paper.

### Conflict of Interest Statement

The authors declare that the research was conducted in the absence of any commercial or financial relationships that could be construed as a potential conflict of interest.
